# Rapid seasonal changes in phenotypes in a wild *Drosophila* population

**DOI:** 10.1038/s41598-023-48571-x

**Published:** 2023-12-19

**Authors:** Takahisa Ueno, Akiko Takenoshita, Kaiya Hamamichi, Mitsuhiko P. Sato, Yuma Takahashi

**Affiliations:** 1 Graduate School of Science, Chiba University, Chiba, Japan; 2 Faculty of Science, Chiba University, Chiba, Japan; 3 Graduate School of Science and Engineering, Chiba University, Chiba, Japan; 4 Department of Frontier Research and Development, Kazusa DNA Research Institute, Kisarazu, Japan

**Keywords:** Genetic variation, Evolutionary ecology

## Abstract

Seasonal environmental change is one of the most rapid and striking environmental variables. Although relatively rapid adaptation to environmental changes over several years or several decades has been described in many taxa, rapid responses to seasonal environments are delicate, and therefore, the detection of the evolutionary responses requires sensitive methods. In this study, we examined seasonal changes in phenotypes related to thermal tolerance and morphological traits of *Drosophila lutescens* collected at the spring and autumn periods from a single location. We first demonstrated that flies in the two seasonal periods were almost genetically identical using double-digest restriction site-associated DNA sequencing and analysis. Using an experimental design to eliminate the effect of possible confounding factors that influence phenotypes (i.e., maternal effects and the environmental conditions in which each phenotype was analyzed), we showed that the heat tolerance of *D. lutescens* was significantly higher in the autumn population than in the spring population. Furthermore, cold tolerance was slightly higher in the spring population than in the autumn one. Although wing length and thorax length did not change significantly between seasons, the ratio of wing length to thorax length changed significantly between them. These results suggest that seasonal environmental heterogeneity induces rapid phenotypic changes within a year. Finally, we discuss the possibility of rapid evolutionary responses to seasonal changes.

## Introduction

Seasonal environmental heterogeneity presents rapid cyclic variation over time. Such environmental heterogeneity can impose highly variable selection on populations and regulate the evolutionary adaptation of species with multiple generations within a year^1–4^. *Drosophila* species are among the most useful species for evaluating rapid seasonal evolution^5,6^. Studies have provided elegant evidence for rapid evolution by establishing isogenic lines^7–10^. In the fruit flies *Drosophila melanogaster* and *D. simulans*, the strains established from females collected at four different seasonal timepoints exhibited markedly different phenotypes with respect to several life-history traits^9^. Another study revealed the evolutionary tracking of various phenotypes in response to seasonal environmental change in a field experiment^11^. These studies suggested the presence of rapid evolutionary responses to seasonal environmental changes. Importantly, almost all previous studies that demonstrated seasonal changes in phenotypes were unable to distinguish between genetically induced and environmentally induced phenotypic changes, because the phenotypes of each seasonal generation were not examined simultaneously (that is, under the same environmental conditions). There is a possibility that experimental (laboratory) conditions are not completely stable and change seasonally (e.g., humidity). A previous study suggested that unknown and/or uncontrollable environmental variation among seasons under laboratory conditions determines the phenotypes expressed, leading to rapid apparent seasonal changes in phenotype^12^. Even under laboratory conditions, evidence of evolution between seasons can be erroneously found if phenotypes are measured in each strain derived from a different season in the seasons corresponding to the derivations. The verification of seasonal rapid evolution in a habitat is delicate and therefore requires elaborate research methods. To obtain evidence of evolution, phenotypic variation should be accurately resolved into genetically induced phenotypic changes and environmentally induced phenotypic changes^12–14^. Therefore, one of the most effective methods to detect genetically induced phenotypic changes is to perform a simultaneous common-garden experiment.

Seasonal changes/oscillations in allele frequency provide additional evidence of seasonal evolution. In *D. melanogaster,* genome-wide population genetic analysis revealed that hundreds of single nucleotide polymorphisms (SNPs) oscillated between spring and fall over multiple years^10^. This result suggests that evolutionary changes may occur across seasons. Importantly here, differences in phenotype and allele frequency among seasons could be generated without seasonal evolution. For example, seasonal changes in phenotype and allele frequency can also be detected when the populations found in different seasons are genetically independent^15,16^ or when seasonal populations are differentially affected by season-specific migration^17,18^. Thus, we must examine whether seasonal populations are genetically identical to verify seasonal evolution in phenotypes.

The present study aimed to perform an appropriate evaluation of rapid phenotypic changes in *D. lutescens* in response to seasonal environmental changes. First, we established two sets of isofemale lines of *D. lutescens* derived from flies collected at two periods, spring and autumn, in a single location. Then, we tested the genetic structure and assessed the genetic differentiation in the two seasonal populations to confirm whether population structures in the two periods were affected by seasonal immigration. Finally, for all strains, we simultaneously measured the thermal tolerance, thorax length, wing length, and wing to thorax ratio.

## Material and methods

### Field collections of study species

*Drosophila lutescens* is a species of the *takahashii* subgroup that is distributed in Korea and Japan^19^. Adults of *D. lutescens* are observed throughout the year in Japan^20^. Our collection site was located in Chiba prefecture, Japan, where the average daily temperature increases in early summer and declines in fall (Fig. S1). The average temperature of the sampling site fluctuated seasonally, between 2.7 and 31.7 °C. For this study, adults were collected on the Nishi-Chiba campus of Chiba University, Japan (35° 37′ 34″ N, 140° 6′ 8″ E), using baited traps during two seasons in 2020: from mid-February to early March (i.e., spring generation) and from early October to early November (i.e., autumn generation). Each collected female was isolated to establish isofemale lines. These siblings were maintained on media contained in plastic vials (φ30 mm × 100 mm) (KFB-1M, Chiyoda Science). The media used was the one described in the study by^21^ (500 ml H_2_O, 50 g sucrose, 25 g active yeast, 8 g agar, 5.36 g KNaC_4_H_4_O_6_·4H_2_O, 0.5 g KH_2_PO_4_, 0.25 g NaCl, 0.25 g MgCl_2_, 0.25 g CaCl_2_, 0.35 g Fe_2_(SO_4_)·6.9H_2_O). The flies were reared under a 12 L:12 D cycle at 25 °C, which are standard conditions for *D. melanogaster*^10,22,23^. In total, 49 and 23 isofemale lines were established for the spring and autumn generations, respectively. All isofemale lines were reared for a minimum of three generations to remove environmental and maternal effects before being used in all experiments described below. All experiments throughout our study were performed using adult females from December 24th, 2020, to February 3rd, 2021. In the assay of heat tolerance and chill coma recovery, only mature females with undamaged wings and a swollen abdomen were selected from rearing vials. This selection was implemented because some previous studies have detected differences in phenotypes when studying only adult females^24–26^. Selected individuals were anaesthetized with CO_2_ and transferred into new vials containing a piece of filter paper soaked in 10% sucrose. Individual flies were allowed to recover for more than two hours in an incubator (12 L:12 D, 25 °C) before initiating the assays.

### Molecular analyses

To compare the population genetic structures of the flies collected in the spring and autumn periods, double-digest restriction site-associated DNA sequencing (ddRAD-seq) was performed. To obtain a high concentration of DNA, genomic DNA was extracted from approximately 10 adult females for each isofemale line using a Maxwell^®^ 16 LEV Plant DNA Kit (cat. #AS1420, Promega) and subsequently fragmented by restriction enzyme digestion using *PstI* and *MspI*. All isofemale lines were sequenced using ddRAD-seq of 100-bp paired-end reads and a DNBSEQ-G400 instrument (MGI Tech. Co., Ltd.). Raw sequence reads were cleaned using Trimmomatic (ver. 0.39). Quality-filtered reads were processed using the *denovo_map.pl* script with the -M 3 option in Stacks v2.53^27^ to reduce sequencing artifacts within the data and allow for SNPs. Only the first SNP per RAD tag was used for population genetic analyses to avoid strong linkage between SNPs. The minor allele frequency threshold was set at 3%, and the missingness by line filter was set at 1%. Based on the filtered SNPs, the population genetic structure was examined using principal component analysis (PCA) in PLINK v1.90. In addition, Wright’s *F*_ST_ between the two periods was calculated using all filtered SNPs and the SambaR package in R version 4.03^28^.

### Heat tolerance assay

Heat tolerance was measured in terms of the heat knockdown time using 5–10 recovered females per isofemale line placed individually in a small plastic tube (12 × 12 × 45 mm) that was sealed with an air-penetrable plug. The containers were tethered in a stainless-steel tube rack placed in a 37 °C water bath. Following the procedure of^25^, the heat knockdown time was scored as the time taken by an individual fly to be knocked down and remain immobilized even after the containers were shaken.

### Chill coma recovery assay

Cold tolerance was measured in terms of chill coma recovery time using 5–10 recovered females per isofemale line. Each female was placed in a small plastic vial (φ15 mm × 95 mm) that was sealed with an air-penetrable plug. Following the procedure of MacMillan et al.^24^, the vials were immersed directly into a 1 L mixture of ice and water in a container (24.5 × 18.5 cm, 10.2 cm in height) at 0 °C, where they remained for 10 min. Individual flies were then quickly transferred into a Petri dish preheated to 25 °C and filmed for 15 min under LED lighting in an incubator (25 °C). The time until the flies started walking was recorded. The recovery time of the individuals that were not walking within 15 min was recorded as 900 s.

### Body size

As proxies for overall body size, thorax length and wing length were quantified in 4 or 5 adult females per isofemale line. Following the procedure of^26^, the length of a wing removed from a female was measured as a straight line drawn from the intersection of the L2 and L3 longitudinal veins to where the L3 longitudinal vein intersected the wing margin. Thorax length was measured from the base of the anterior humeral bristle to the posterior tip of the scutellum. In addition, as an index of dispersal ability, the wing to thorax ratio was calculated as wing length/thorax length.

The broad-sense heritability (*H*^2^)^29^ was estimated by calculating the variance components of a between-isofemale line and a within-isofemale line using one-way ANOVA in the *VCA* package in R.

### Statistical analyses

All analyses were conducted in R version 4.03. Differences between seasons were analyzed by linear mixed models (LMMs) for body size and generalized linear mixed models (GLMMs) for thermal tolerance (gamma distribution) in the *lme4* package. In both mixed models, isofemale lines and experimental assay dates were treated as random effects to consider in the pseudo-replication of individuals within isofemale lines. The *P* values of the fixed effects in the LMM and GLMM were calculated using the χ^2^ test of the *car* package in R. In addition, the effect sizes (Cohen’s *d*) were calculated to compare wing length, thorax size, and wing to thorax ratio.

## Results

**Figure 1:**
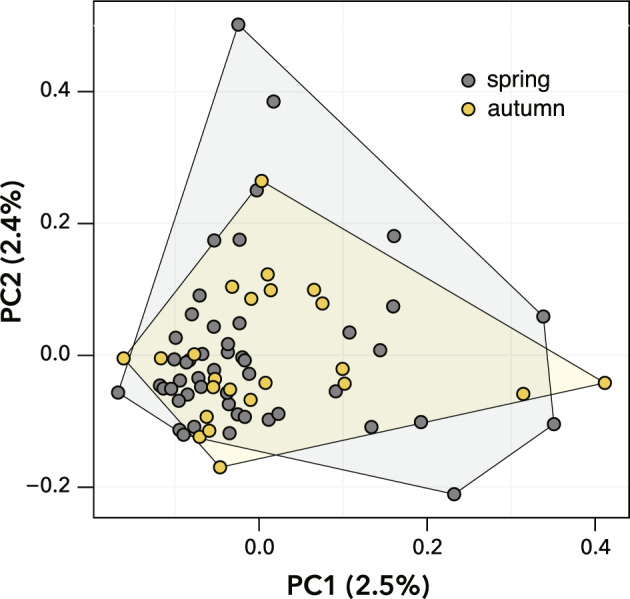
Representation of *Drosophila lutescens* collected in spring and autumn via principal component analysis (PCA). Convex polygons for each period are shown in the two-dimensional space. Dots represent isofemale lines (*N* = 72; spring: 49, autumn: 23).

Information on the number of raw reads for each isofemale line is summarized in Table S1. A total of 1828 SNP loci were shared among the isofemale lines. Using these loci, PCA was performed to identify the relationship between the two seasons. Two-dimensional plots of PC1 and PC2 showed that isofemale lines derived from the two seasons overlapped (Fig. 1). Using all SNPs, the *F*_ST_ value between the spring and autumn periods was estimated to be 0.007.

**Figure 2:**
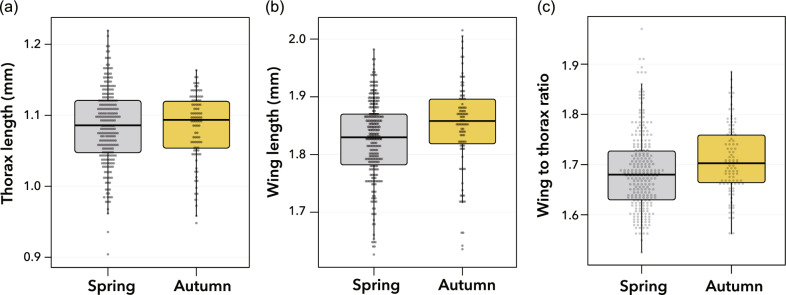
Body size of *Drosophila lutescens* from the spring and autumn periods*.* Thorax length (**a**), wing length (**b**), and wing-to-thorax ratio (**c**). In all boxplots, boxes and thick black lines represent the 25th (lower) to 75th (upper) percentile and median, respectively. The upper and lower whiskers represent scores outside the middle 50%. The plots show all sample data.

There was no significant difference in the thorax length of the adult females between the spring and autumn periods (Fig. 2a; χ^2^ = 0.21, *df* = 1, *P* = 0.65, Cohen’s *d* =  − 0.03). The wing length of females from the autumn period was slightly longer than that from the spring period, but the difference was not statistically significant (Fig. 2b; χ^2^ = 3.08, *df* = 1, *P* = 0.08, Cohen’s *d* = 0.34). However, the females from the autumn period had a significantly greater wing-to-thorax ratio than those from the spring period (Fig. 2c; χ^2^ = 4.67, *df* = 1, *P* = 0.03, Cohen’s *d* = 0.33). The *H*^2^ values of thorax length, wing length, and wing to thorax ratio were 0.17, 0.42, and 0.24, respectively.

**Figure 3:**
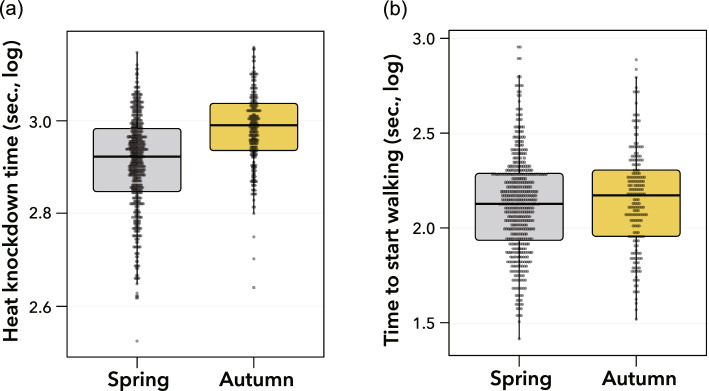
Thermal tolerance of *Drosophila lutescens* from the spring and autumn periods to heat (**a**) and cold (**b**). The meaning of the boxes, thick black lines, and points is described in Fig. 2.

In the heat tolerance assay, females often remained on the tube wall or stood on the tube bottom prior to heating. Upon transfer to a preheated water bath, the females dropped to the bottom of the tube and were immobilized within an average of 876 s (minimum: 335 s, maximum: 1441 s). The heat knockdown time for flies from the autumn period was significantly longer than that for flies from the spring period (Fig. 3a; χ^2^ = 18.67, *df* = 1, *P* < 0.001). On the other hand, in the chill coma recovery assay, females immersed in an ice bath started to walk ca. 220 s on average after being transferred to a 25 °C incubator. Although the time the flies took to start walking tended to be shorter for flies from the spring period than for those from the autumn period, the difference in the recovery time was not significant between the two groups (Fig. 3b; χ^2^ = 0.18, *df* = 1, *P* = 0.67). The *H*^*2*^ values of heat knockdown time and chill coma recovery were 0.26 and 0.06, respectively.

## Discussion

Differences in traits across seasons are known to occur in various organisms^30,31^. This phenomenon is suggested to be evidence supporting rapid evolution. However, comparing phenotypes in each season alone is not sufficient to prove rapid evolution among seasons. The effects of plasticity and the season-specific migration of populations with different genotypes can also explain the observed seasonal changes. One of the most important steps to detect rapid evolution in a natural population is improving the method for measuring phenotypic values in each season. The present study first demonstrated that the *F*_ST_ values between the spring and autumn periods of *D. lutescens* were very low; the two periods exhibited little genetic differentiation, and therefore, season-specific migration is unlikely to occur in the population that we observed. Nevertheless, we detected significant differences in heat tolerance and the wing to thorax ratio between the two collection time periods. Removing environmental effects, which are potential factors affecting phenotypic values, allowed for strong confirmation of the presence of genetic changes across seasons. Therefore, as in previous studies, this difference may reflect rapid seasonal evolution. It should be noted that flies collected in the spring period were kept in our laboratory for a longer time than flies collected in the autumn period. Unfortunately, we cannot currently exclude the possibility that a difference in breeding length in the laboratory affects the phenotypic values owing to inbreeding depression, stochastic drift, or adaptation to laboratory conditions^32^. To mitigate these effects, we should rear our lines for sufficiently long before phenotyping or reverse the order of seasonal sampling. Detecting oscillations in phenotypic values over several years is also important to provide evidence to support rapid evolution. In addition, simultaneous measurements of phenotypes should be undertaken at multiple sites to deepen the understanding of rapid seasonal evolution.

A higher heat tolerance after summer (i.e., autumn period) suggests that selection associated with seasonal climate drives the adaptive evolutionary response. The prevalence of individuals with higher heat tolerance might increase in the population during summer and decrease during winter. Although the heritability estimates of heat tolerance among studies may not be directly comparable because of the different assessments used, our estimate of the heritability of heat tolerance was similar to that reported in^33^, whereas our heritability estimate of cold tolerance was very low compared with previously reported heritability^34^. In the population studied in the present study, the genetic variation in heat tolerance, but not cold tolerance, may be maintained across seasons in our specific population of *D. lutescens*.

Insects often show latitudinal variation in flight morphology, such as wing size and the ratio of wing size to body size, which directly affects foraging, mating, dispersal, and thus reproductive success. In the present study, the effect size (Cohen’s *d*) showed that the difference in wing size was greater than that in thorax size. Therefore, the difference in ratio of wing to thorax may be attributed to the difference in wing size. Along the environmental gradient on a continental scale, the wing size relative to body size of *Drosophila* spp. is known to be larger in cold regions than in warm regions. Larger wings are advantageous in the cold because ectotherms generate less energy per wingbeat^35^. However, the opposite pattern was observed for the wing-to-thorax ratio in the context of seasonal environmental changes; a larger ratio of wing-to-thorax length was observed in flies from the autumn period, which had just experienced summer. Such an opposite pattern could be explained by seasonal variation in population density. Previous theoretical studies demonstrated that a dispersal strategy could evolve in a density-dependent manner^36^. The wing to thorax ratio could reflect the ability to access resources; that is, a larger wing to thorax ratio could increase dispersal ability^37^. Since the population density of *Drosophila* spp. could be higher during a warm season than during a cold season, genotypes expressing a higher dispersal ability (i.e., larger wing to thorax ratio) may be favoured during summer, when densities and competition increase.

### Supplementary Information

Supplementary Information.

## Data Availability

Phenotypic and SNP data of ddRAD-seq are available in the publicly accessible repository Dryad at https://doi.org/10.5061/dryad.kh189327r. (temporal link for reviewers: https://datadryad.org/stash/share/6AbpZnbLna5Ilig_tU0IpUIpRQc-lHuR9yTcRbTRbVo).
