# Supplementary material for: Rapid seasonal changes in phenotypes in a wild Drosophila population
Source: Sci Rep. 2023 Dec 19;13:21940. doi: 10.1038/s41598-023-48571-x (PMC10730618; doi:10.1038/s41598-023-48571-x)
Supplement: Supplementary file 1 — Supplementary Information. [file 41598_2023_48571_MOESM1_ESM.docx]

**Appendix**

Appropriate evaluation of rapid evolutionary responses to seasonal environmental variability in a wild *Drosophila* population

Takahisa Ueno^1^, Akiko Takenoshita^2^, Kaiya Hamamichi^2^, Mitsuhiko P. Sato^3^ and Yuma Takahashi^4^

1. Graduate School of Science and Engineering, Chiba University, Chiba, Japan

2. Faculty of Science, Chiba University, Chiba, Japan

3. Department of Frontier Research and Development, Kazusa DNA Research Institute

4. Graduate School of Science, Chiba University, Chiba, Japan

orresponding author: Yuma Takahashi (E-mail: takahashi.yum@gmail.com)

**Table S1.** The number of raw reads in each isofemale line.

| Isofemale line No. | Seasonal period | Reads |
| --- | --- | --- |
| 15 | Spring | 3338867 |
| 23 | Spring | 3733218 |
| 24 | Spring | 3424709 |
| 25 | Spring | 2896408 |
| 26 | Spring | 3516919 |
| 27 | Spring | 3857630 |
| 28 | Spring | 3707780 |
| 29 | Spring | 3133594 |
| 31 | Spring | 3203799 |
| 39 | Spring | 4135463 |
| 40 | Spring | 2984048 |
| 42 | Spring | 3454823 |
| 44 | Spring | 2845660 |
| 49 | Spring | 3348005 |
| 50 | Spring | 3829729 |
| 51 | Spring | 3108543 |
| 53 | Spring | 3285473 |
| 54 | Spring | 3767425 |
| 55 | Spring | 3776354 |
| 58 | Spring | 2915372 |
| 59 | Spring | 3130028 |
| 66 | Spring | 3503538 |
| 75 | Spring | 4049027 |
| 77 | Spring | 3335159 |
| 78 | Spring | 3005131 |
| 79 | Spring | 3461525 |
| 81 | Spring | 2981003 |
| 82 | Spring | 3722754 |
| 83 | Spring | 3584832 |
| 84 | Spring | 4486455 |
| 85 | Spring | 3231009 |
| 88 | Spring | 3132774 |
| 91 | Spring | 4174406 |
| 97 | Spring | 4706774 |
| 98 | Spring | 3585392 |
| 99 | Spring | 3915297 |
| 101 | Spring | 4021596 |
| 102 | Spring | 4724621 |
| 104 | Spring | 2744121 |
| 106 | Spring | 2660050 |
| 110 | Spring | 3038443 |
| 113 | Spring | 3507002 |
| 114 | Spring | 2545051 |
| 120 | Spring | 3193415 |
| 122 | Spring | 3115692 |
| 130 | Spring | 3303736 |
| 131 | Spring | 4074485 |
| 135 | Spring | 2640265 |
| 137 | Spring | 2714557 |
| 355 | Autumn | 3945930 |
| 384 | Autumn | 3141066 |
| 385 | Autumn | 3745097 |
| 387 | Autumn | 3974438 |
| 389 | Autumn | 2909073 |
| 500 | Autumn | 2687524 |
| 508 | Autumn | 3018026 |
| 510 | Autumn | 3434852 |
| 531 | Autumn | 3729816 |
| 533 | Autumn | 3230959 |
| 535 | Autumn | 2896025 |
| 538 | Autumn | 3278880 |
| 539 | Autumn | 4400868 |
| 541 | Autumn | 2901996 |
| 543 | Autumn | 3382202 |
| 544 | Autumn | 3372686 |
| 547 | Autumn | 3623843 |
| 551 | Autumn | 3254899 |
| 552 | Autumn | 3415532 |
| 555 | Autumn | 3373317 |
| 556 | Autumn | 3475196 |
| 581 | Autumn | 3559574 |
| 591 | Autumn | 3232658 |


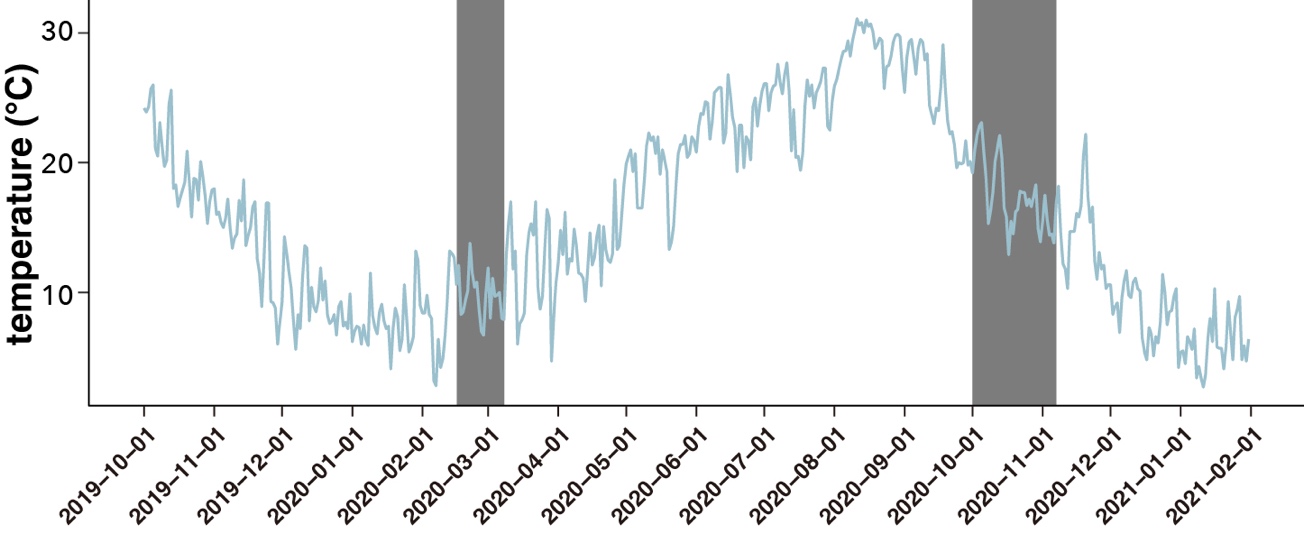


**Figure S1.** Average daily temperature in Chiba from 1 October 2019 to 31 January 2021. This data was published by the Japan Meteorological Agency. Grey bands indicate our collection periods.
